# Prediction Models of Cognitive Trajectories in Patients with Nonamnestic Mild Cognitive Impairment

**DOI:** 10.1038/s41598-018-28881-1

**Published:** 2018-07-11

**Authors:** Jin San Lee, Seong-Kyung Cho, Hee Jin Kim, Yeo Jin Kim, Key-Chung Park, Samuel N. Lockhart, Duk L. Na, Changsoo Kim, Sang Won Seo

**Affiliations:** 1 Department of Neurology, Samsung Medical Center, Sungkyunkwan University School of Medicine, Seoul, 06351 Korea; 2 Neuroscience Center, Samsung Medical Center, 06351 Seoul, Korea; 3 Department of Neurology, Kyung Hee University Hospital, Seoul, Korea; 4 Department of Preventive Medicine, Yonsei University Wonju College of Medicine, Wonju, Korea; 5 Department of Neurology, Chuncheon Sacred Heart Hospital, Hallym University College of Medicine, Chuncheon, Korea; 6 Department of Internal Medicine, Wake Forest School of Medicine, Winston-Salem, USA; 7 Department of Preventive Medicine, Yonsei University College of Medicine, Seoul, Korea; 8 Department of Health Sciences and Technology, SAIHST, Sungkyunkwan University, Seoul, 06351 Korea; 9 Clinical Research Design and Evaluation, SAIHST, Sungkyunkwan University, Seoul, 06351 Korea

## Abstract

To evaluate prediction models of cognitive trajectories in patients with nonamnestic mild cognitive impairment (naMCI) using group-based trajectory analysis, we evaluated 121 patients with naMCI who underwent at least their first three yearly assessments. Group-based trajectory models were used to classify cognitive trajectories based on Clinical Dementia Rating Sum of Boxes scores over four years in patients with naMCI. A total of 22 patients (18.2%) were classified into the “fast-decliners” group, while 99 patients (81.8%) were classified into the “slow-decliners” group. The mean age was higher in the fast-decliners than in the slow-decliners (*p* = 0.037). Compared to the slow-decliners, the fast-decliners were more frequently impaired in the domains of language (*p* = 0.038) and frontal/executive functions (*p* = 0.042), and had more frequent multiple-domain cognitive impairment (*p* = 0.006) on baseline neuropsychological tests. The rate of conversion to dementia was significantly higher in the fast-decliners than in the slow-decliners (86.4% vs. 10.1%, *p* < 0.001). Our findings showed that there are indeed distinct patterns of cognitive trajectories in patients with naMCI. Close observation of naMCI patients’ baseline demographic and clinical profiles in clinical settings may help identify individuals at greatest risk for dementia.

## Introduction

Mild cognitive impairment (MCI) is a clinically heterogeneous syndrome, and the MCI syndrome can be classified into amnestic and nonamnestic MCI (naMCI) subtypes depending on the degree of impairment in the memory domain^1^. Patients with naMCI have impairments in other cognitive domains than memory (e.g. frontal/executive, language, or visuospatial). Previous studies have shown that amnestic MCI patients have a high likelihood of progressing to Alzheimer’s Disease (AD) dementia, whereas naMCI patients have a higher likelihood of progressing to a non-AD dementia^2–5^. In particular, the causes and outcomes of cognitive impairments in naMCI may be more heterogeneous^6^. However, to date, the long-term cognitive trajectories in patients with naMCI, assessed using clinical and functional measures, are not well known.

Many studies have examined the clinical and neuropsychological profiles related to the likelihood of progression from MCI to dementia. These previous reports have shown that older age, verbal memory impairment, frontal/executive dysfunction, multiple-domain impairment, and the presence of at least one apolipoprotein E (*APOE)* ε4 allele increase the risk of conversion to dementia^7–12^. However, most previous studies have evaluated clinical outcomes by comparing naMCI with amnestic MCI, or by combining naMCI and amnestic MCI patients together^12–14^. So far, there have been no studies investigating the clinical profiles related to disease progression of naMCI separately from amnestic MCI. Since the research criteria for MCI due to AD consider both amnestic MCI and naMCI as possible prodromal stages of AD-type dementia, understanding the cognitive trajectories of naMCI has clinical importance^15^.

Group-based trajectory analysis provides a tool for figuratively painting a statistical portrait of the predictors and consequences of distinct trajectories of development^16^. It also can enable identification, summarization, and communication of complex patterns in longitudinal data^17^. Group-based models have been applied to address questions related to developmental trajectories in psychology^18,19^, medicine^20^, and criminology^21^. Several studies have also used these models to facilitate causal inference in situations where randomization to treatment condition is not possible^22,23^. Recently, a few studies have used trajectory analysis to identify predictive or prognostic factors in patients with MCI^10,24,25^. However, no studies have applied this method to naMCI patients to determine longitudinal cognitive trajectories.

To better understand the cognitive trajectories of naMCI, we evaluated 121 patients with naMCI who underwent at least their first three yearly assessments. The primary goal was to classify longitudinal cognitive trajectories of naMCI using group-based trajectory analysis. The secondary goal was to evaluate the demographic and clinical risk factor profiles which best predicted the prognosis of naMCI patients. We hypothesized not only that group-based trajectory analysis would enable to identification of distinct groups of naMCI patients based on longitudinal trajectories of decline, but also that specific patient characteristics would predict membership in these naMCI trajectory groups.

## Results

### Demographic and clinical characteristics

The demographic and clinical characteristics of participants at baseline are presented in Table 1. The mean age of participants was 71.0 years and 87 (71.9%) were female. *APOE* genotyping was performed in 76 (62.8%) of 121 patients, and 14 were ε4 carriers (11.6% of all patients). The mean (standard deviation [SD]) Mini-Mental Status Examination (MMSE), Clinical Dementia Rating (CDR), and Clinical Dementia Rating Sum of Boxes (CDR-SB) scores at baseline were 26.2 (3.5), 0.5 (0.1), and 1.3 (0.9), respectively. The most frequently involved cognitive domain was frontal/executive function (45.5%), followed by language (43.8%) and visuospatial (25.6%) functions. A total of 28 (23.1%) patients were impaired in multiple cognitive domains; of these, five (17.9%) had three domains affected and 23 (82.1%) had two domains involved.

**Table 1:** Demographic and clinical characteristics of the study participants.

	Total (N = 121)
Age, years	71.0 (7.3)
Female, N (%)	87 (71.9)
Education, years	9.0 (5.3)
Total follow-up, years	3.8 (0.8)
*APOE4* carrier, N (%)*	14 (11.6)
Vascular risk factors	
Hypertension, N (%)	60 (49.6)
Diabetes, N (%)	39 (32.2)
Dyslipidemia, N (%)	32 (26.4)
Cardiovascular disease, N (%)	21 (17.4)
History of stroke, N (%)	11 (9.1)
GDepS	15.0 (8.4)
Range	0–24
MMSE	26.2 (3.5)
Range	22–30
CDR	0.5 (0.1)
Range	0–0.5
CDR-SB	1.3 (0.9)
Range	0–2.5
Involved cognitive domain	
Attention	28 (23.1)
Language	53 (43.8)
Visuospatial	31 (25.6)
Frontal/executive	55 (45.5)
Multiple-domain	28 (23.1)

Values are mean (SD), score, or N (%).

*APOE genotyping was performed in 76 (62.8%) of the 121 patients with naMCI.

Abbreviations: N = number; SD = standard deviation; *APOE4* = apolipoprotein E ε4; GDepS = Geriatric Depression Scale, MMSE = Mini-Mental State Examination; CDR = Clinical Dementia Rating; CDR-SB = Clinical Dementia Rating Scale Sum of Boxes.

### Groups identified by group-based trajectory analysis

**Figure 1:**
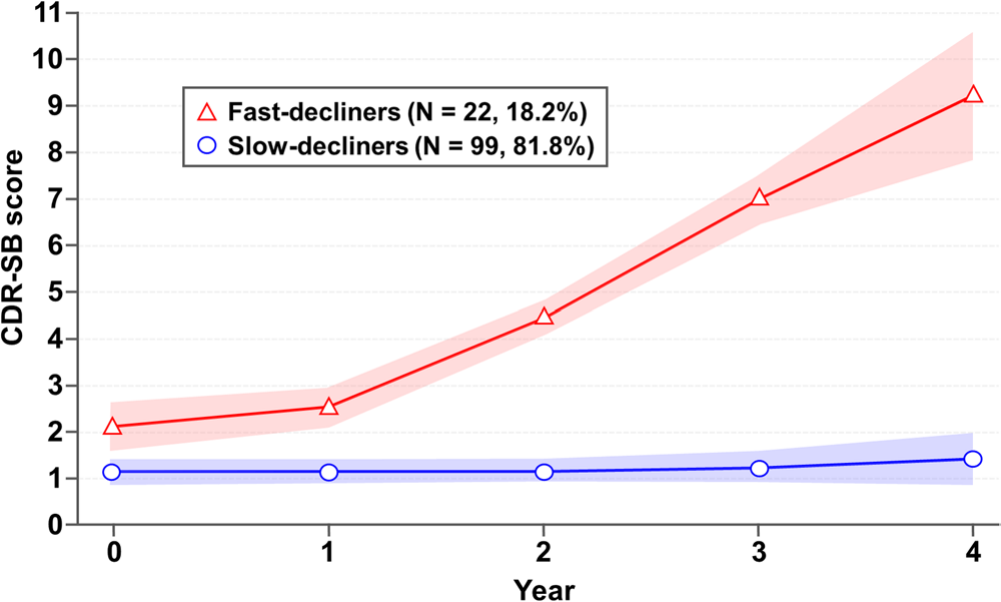
Cognitive trajectories based on CDR-SB score from baseline (year 0) to year 4 among 121 patients with naMCI. Shown are results of the group-based trajectory analysis used to identify groups of patients following a similar trajectory of cognitive decline over time, as assessed with the CDR-SB score at years 0, 1, 2, 3, and 4. The 2 cognitive trajectories that were identified are shown with 95% confidence intervals (red or blue shaded area). CDR-SB = Clinical Dementia Rating Scale Sum of Boxes; naMCI = nonamnestic mild cognitive impairment.

In order to define the cognitive trajectories based on CDR-SB score in patients with naMCI, we tested solutions varying the number of groups from one to four. According to the Bayesian Information Criterion (BIC) value (−698.08), the two-group solution with a quadratic polynomial shape was found to be the most appropriate. The cognitive trajectories in 121 patients with naMCI are illustrated in Fig. 1. A total of 22 patients (18.2%) were classified into the “fast-decliners” group, while 99 patients (81.8%) were classified into the “slow-decliners” group. The change in CDR-SB score over four years was greater than seven points in the fast-decliners, but less than one point in the slow-decliners.

### Identifying variables that distinguish trajectories

**Figure 2:**
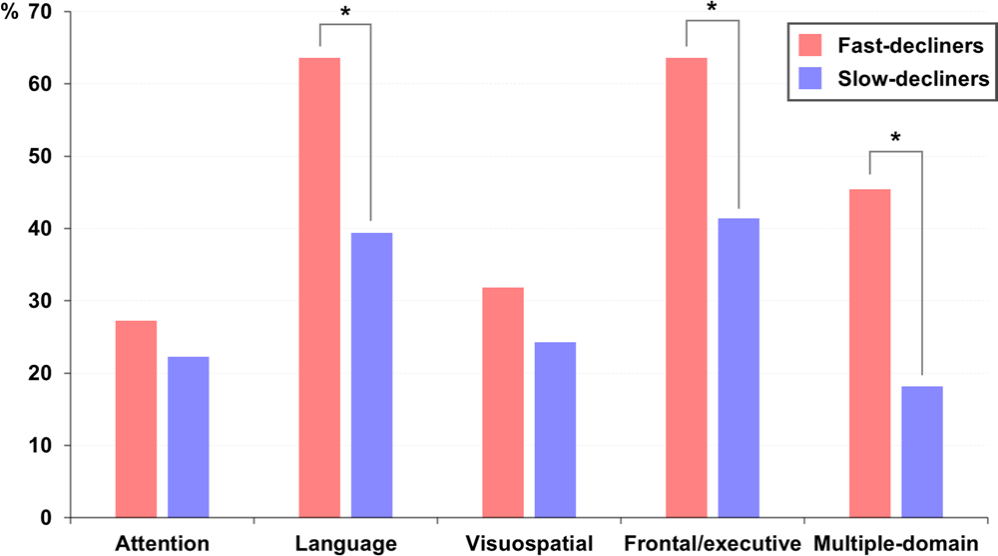
Comparisons between proportions of naMCI patients in the fast-decliners (red) and slow-decliners (blue) groups with abnormal baseline neuropsychological test results. naMCI = nonamnestic mild cognitive impairment. *p < 0.05.

Table 2 presents the results of comparisons of demographic and clinical characteristics between the fast- and slow-decliners. The mean age was higher in the fast-decliners than in the slow-decliners (74.0 ± 6.7 vs. 70.4 ± 7.3, *p* = 0.037). There were no differences in the ratios of gender and years of education between the fast- and slow-decliners. In the fast-decliners, the cognitive and functional impairment evaluated by the CDR-SB at baseline was significantly greater than that of the slow-decliners (2.1 ± 0.8 vs. 1.1 ± 0.7, *p* < 0.001). In addition, we compared the proportion of participants with abnormal results on baseline neuropsychological tests between the two groups. Compared to the slow-decliners, the fast-decliners were impaired more frequently in the domains of language (*p* = 0.038) and frontal/executive functions (*p* = 0.042), and had more frequent multiple-domain cognitive impairment (*p* = 0.006, Fig. 2). In addition, logistic regression analysis controlling for age and the number of impaired cognitive domains confirmed that impaired language (OR 7.7, *p* = 0.046) and frontal/executive functions (OR 8.2, *p* = 0.040) significantly predicted membership in the fast-decliners group.

**Table 2:** Comparisons of demographics, baseline neuropsychological test performances, and dementia conversion between the fast- and slow-decliners in patients with naMCI.

	Fast-decliners (N = 22)	Slow-decliners (N = 99)	*P*-value
Age, years	74.0 (6.7)	70.4 (7.3)	0.037
Female, N (%)	18 (81.8)	69 (69.7)	0.253
Education, years	7.7 (5.8)	9.3 (5.2)	0.205
Total follow-up, years	3.5 (0.7)	3.9 (1.4)	0.049
*APOE4* carrier, N (%)	3 (13.6)	11 (11.1)	0.634
Vascular risk factors			
Hypertension, N (%)	13 (59.1)	47 (47.5)	0.324
Diabetes, N (%)	10 (45.5)	29 (29.3)	0.142
Dyslipidemia, N (%)	4 (18.2)	28 (28.3)	0.331
Cardiovascular disease, N (%)	2 (9.1)	19 (19.2)	0.258
History of stroke, N (%)	1 (4.5)	10 (10.1)	0.412
GDepS	16.2 (8.1)	14.8 (8.5)	0.482
CDR-SB	2.1 (0.8)	1.1 (0.7)	<0.001
Conversion to dementia	19 (86.4)	10 (10.1)	<0.001
AD	14 (73.7)	6 (60.0)	
DLB	2 (10.5)	3 (30.0)	
SVaD	2 (10.5)	1 (10.0)	
CBS	1 (5.3)	0 (0.0)	

Values are mean (SD) or number (%). Chi-square and Student’s *t*-tests were performed to compare demographic variables between the fast- and slow-decliners groups.

Abbreviations: N = number; SD = standard deviation; *APOE4* = apolipoprotein E ε4; GDepS = Geriatric Depression Scale, MMSE = Mini-Mental State Examination; CDR = Clinical Dementia Rating; CDR-SB = Clinical Dementia Rating Scale Sum of Boxes; K-BNT = Korean version of the Boston Naming Test; RCFT = Rey-Osterrieth Complex Figure Test; SVLT = Seoul Verbal Learning Test; COWAT = Controlled Oral Word Association Test; AD = Alzheimer’s disease; DLB = dementia with Lewy bodies; SVaD = subcortical vascular dementia; CBS = corticobasal syndrome.

### Longitudinal changes in neuropsychological test performance over time by group-based trajectories

Table 3 shows mixed effects models examining how worsening in performance on neuropsychological tests over time was related to naMCI patient group status, defined by the group-based trajectory method. Significant group-by-time interactions were obtained for most neuropsychological tests from baseline to year five. Over time, the fast-decliners showed significantly worse performance than the slow-decliners in the Korean version of the Boston Naming Test (K-BNT), the Seoul Verbal Learning Test (SVLT), the Rey-Osterrieth Complex Figure Test (RCFT), the Controlled Oral Word Association Test (COWAT, animal), MMSE, CDR, and CDR-SB scores. As shown in Table 2, the rate of conversion to dementia was significantly higher in the fast-decliners (86.4%) than in the slow-decliners (10.1%).

**Table 3:** Mixed effects models of worsening in performance on neuropsychological tests over time by group-based trajectories in patients with naMCI.

Group by time	Fast-decliners vs. Slow-decliners		
	Estimate	SE	*p*-value
Neuropsychological tests			
Digit span: Forward	−0.21	0.09	0.015
Digit span: Backward	−0.10	0.12	0.368
K-BNT	−0.72	0.18	<0.001
RCFT: Copy	−0.51	0.18	0.005
SVLT: Immediate recall	−0.38	0.11	0.001
SVLT: Delayed recall	−0.48	0.14	0.001
SVLT: Recognition score	−0.34	0.12	0.007
RCFT: Immediate recall	−0.37	0.09	<0.001
RCFT: Delayed recall	−0.30	0.09	0.001
RCFT: Recognition score	−0.55	0.18	0.003
Stroop color reading	−0.20	0.12	0.109
COWAT: Animal	−0.29	0.09	0.001
COWAT: Supermarket	−0.21	0.11	0.056
COWAT: Phonemic total	−0.11	0.10	0.305
MMSE	−0.91	0.18	<0.001
CDR	0.19	0.02	<0.001
CDR-SB	1.53	0.12	<0.001

Linear mixed effects model were performed using group (fast-decliners vs. slow-decliners), time, and the interaction term between group and time (group by time) as fixed effects and patient as a random effect. Age-, sex-, and education-specific z-scores were used in the comparison of longitudinal neuropsychological performance between the fast- and slow-decliners groups.

Abbreviations: naMCI = non-amnestic mild cognitive impairment; SE = standard error; K-BNT = Korean version of the Boston Naming Test; RCFT = Rey-Osterrieth Complex Figure Test; SVLT = Seoul Verbal Learning Test; COWAT = Controlled Oral Word Association Test; MMSE = mini-mental state examination; CDR = Clinical Dementia Rating; CDR-SB = Clinical Dementia Rating sum of boxes.

## Discussion

We assessed longitudinal cognitive trajectories in a sample of 121 patients with naMCI using group-based trajectory analysis based on CDR-SB scores. The results from the present study showed that there are indeed distinct patterns of cognitive trajectories in patients with naMCI: one group can be classified as fast-decliners, while the remaining participants can be classified as slow-decliners. Furthermore, we found that several baseline demographic and clinical characteristics, including older age and impairments in the cognitive domains of language and frontal/executive functions, are risk factors for predicting future decline among naMCI patients. Given the increasing interest in the clinical outcomes of naMCI, our results suggest that close observation of naMCI patients’ baseline demographic and clinical profiles may help identify individuals at greatest risk for dementia.

The novelty of the present study is that it takes into account the variability of cognitive trajectories based on CDR-SB in individual patients, and thereby seeks to refine our understanding of the relationship between disease progression and prognosis. We found that the trajectory groups derived from our group-based trajectory modeling were a convenient statistical device for understanding cognitive trajectories in patients with naMCI. The fast-decliners, representing less than 20% of the study participants, showed continuous deterioration in CDR-SB scores over time, while the slow-decliners showed no change. To date, only a few studies have investigated longitudinal performance in patients with naMCI^26^. However, these previous studies operationally defined clinical progression as a worsening on the CDR-SB over two years. In contrast, the trajectory analysis performed in the present study was designed to identify clusters of individuals who have followed a similar developmental trajectory on an outcome of interest^17,27,28^. To the best of our knowledge, this is the first study to conduct a data-driven classification of naMCI patients based on longitudinal performance, rather than a longitudinal analysis based on *a priori* classification.

Another noteworthy finding was that there were distinct baseline demographic and clinical profiles able to predict the prognosis of patients with naMCI. At baseline evaluation, the fast-decliners were older, had more frequent impairments in the domains of language and frontal/executive functions, and had more frequent multiple-domain involvement on neuropsychological testing compared to the slow-decliners. Our finding is partly consistent with a previous study, which showed that naMCI patients who clinically progressed were older and had lower baseline performance on category fluency and visuospatial tasks compared with naMCI patients who remained stable^26^. That study sample, however, consisted of single-domain (frontal/executive dysfunction) naMCI patients. Notably, we found baseline impairments in both language and frontal/executive functions in patients with naMCI strongly predicted membership in the fast-decliners group, even after controlling for age and the number of involved cognitive domains. Our results therefore suggest that investigating baseline clinical profiles in patients with naMCI has important implications for identifying individuals who are at risk for dementia.

In the present study, mixed effects models also demonstrated that the fast-decliners group exhibited worsening in neuropsychological test performance over time, including worsening in the memory domain. Indeed, patients with naMCI in our study mainly converted to clinically diagnosed AD dementia. Our findings are not in line with some previous studies showing that naMCI patients have a higher likelihood of progressing to a non-AD dementia^2–5^. This discrepancy could be explained by factors including differences between clinical and pathological diagnosis, a relatively small sample size, or insufficient follow-up. Indeed, several other studies have shown that a substantial number of naMCI patients progress to AD dementia during follow-up^29,30^.

Several limitations of the present study should be acknowledged. First, as the number of patients in the fast-decliners group was relatively small, the analyses may have had low statistical power. However, considering the prevalence of naMCI in community-based studies (0.5–6%)^13,31,32^, the results of our study still have clinical significance. Further investigation with larger sample sizes and a longer follow-up period is needed to understand long-term trajectory of naMCI. Second, it is not a population-based study, which limits its generalizability to the general population. Third, we did not have molecular imaging or neuropathologic data on the participants. Fourth, there could be a large number of situations where a common pattern of change over time cannot be assumed. Fifth, although we followed naMCI patients for a minimum of three years, this may not have been a sufficient length of time to properly evaluate the likelihood of conversion to dementia. Finally, some of the participants in this study had somewhat high Geriatric Depression Scale (GDepS) or low MMSE scores. However, depression is common in individuals with MCI^33,34^ and the naMCI patients with high GDepS score in this study did not meet the diagnostic criteria for major depressive disorder on baseline screening. Also, many elderly people in South Korea have low educational levels due to the Korean War, thus, the MMSE scores of the elderly who have normal cognitive function are relatively lower than those of other countries. We excluded participants who met criteria for dementia based on the Diagnostic and Statistical Manual of Mental Disorders-IV (DSM-IV)^35^, not on the MMSE score.

In conclusion, our findings showed that there are indeed distinct patterns of cognitive trajectories in patients with naMCI. We suggest that evaluating baseline clinical profiles as risk factors for cognitive deterioration may help inform early-life interventions in patients with naMCI. Close observation of naMCI patients’ baseline demographic and clinical profiles in clinical settings may help identify individuals at greatest risk for dementia.

## Methods

### Participants

The study participants were included from two registry studies: 54 patients with naMCI from the Memory Disorder Clinic in Samsung Medical Center (May 2003 to June 2015) and 67 patients with naMCI from the Clinical Research Center for Dementia of South Korea study, a nationwide multicenter cohort study of cognitive disorders involving 31 memory disorder clinics at universities and general hospitals in South Korea (September 2005 to August 2012)^36^. These two studies used a common standardized diagnostic assessment, which included an assessment for the diagnostic criteria for naMCI. All participants met the clinical criteria proposed by Petersen^1^: cognitive complaints reported by patients or by their caregiver; scores lower than −1.0 SD of the age-, sex-, and education-adjusted norms on tests for at least one of the main cognitive domains except memory; generally intact activities of daily living; and the absence of dementia. They also had completed at least their first three yearly assessments, with the same interview and neuropsychological testing conducted at both their baseline and follow-up evaluations. The study participants were recruited according to the modified questionnaire of Health screening and random recruitment for cognitive aging research^37^.

Participants were excluded if they met criteria for dementia based on the DSM-IV; had a history of a neurological disorder, current psychiatric illness, substance abuse, or head trauma with loss of consciousness; had uncontrolled diabetes or hypothyroidism; or were taking medications that affect cognition. Participants underwent a brain magnetic resonance imaging scan and were excluded if they had a cerebral, cerebellar, or brainstem infarction; hemorrhage; brain tumor; hydrocephalus; severe cerebral white matter hyperintensities (deep white matter ≥2.5 cm and caps or band ≥1.0 cm); or severe head trauma.

### Standard protocol approvals, registrations, and patient consents

We obtained written informed consent from each patient. This study was approved by the Institutional Review Board at the Samsung Medical Center. All methods were carried out in accordance with approved guidelines.

### Neuropsychological testing and clinical assessments

All participants underwent neuropsychological testing using a standardized neuropsychological battery, the Seoul Neuropsychological Screening Battery^38^. The battery contains tests for attention, language, praxis, elements of Gerstmann syndrome, visuoconstructive function, verbal and visual memory, and frontal/executive function. The battery in the present study includes Digit span (forward and backward), K-BNT, RCFT (copying, immediate and 20-minute delayed recall, and recognition), SVLT (3 learning-free recall trials of 12 words, a 20-minute delayed recall trial for these 12 items, and a recognition test), phonemic and semantic COWAT, and a Stroop Test (word and color reading of 112 items during a 2-minute period). Age-, sex- and education-specific norms for each test, based on 447 normal subjects, were used for comparison. Z-scores lower than −1.0 SD of the age-, sex- and education-adjusted norms were considered abnormal. We also performed MMSE, CDR, CDR-SB, and GDepS.

### Conversion to dementia

The diagnosis of dementia was based on criteria from the DSM-IV and required clinical evidence of cognitive deficits confirmed by neuropsychological testing, as well as evidence of impairment in social or occupational functions confirmed by activities of daily living scales. The MMSE or CDR-SB scores were not used in the determination of the diagnosis of dementia. For the diagnosis of probable AD, we used the criteria of the National Institute of Neurological and Communicative Disorders and Stroke-Alzheimer’s Disease and Related Disorders Association^39^. Established diagnostic criteria for clinical dementia with Lewy bodies, subcortical vascular dementia, and corticobasal syndrome were also used^40–42^.

### Statistical analyses

We used group-based trajectory models (SAS Proc Traj; SAS Institute Inc) to classify cognitive trajectories based on CDR-SB scores over four years in 121 patients with naMCI^28^. The model selection for the trajectory analysis was based on the BIC values between models^28^. The BIC enables a balance of both model complexity and model fit, in a manner similar to the adjusted *R*^2^, with lower numbers indicating a better model fit. The appropriate number of trajectories and trajectory shape were selected by recommended procedures^19,28,43^.

Continuous variables were presented as means ± SD and were compared using Student’s *t*-test. Categorical variables were compared using the Chi-square test or Fisher’s exact test. To investigate the effects of baseline neuropsychological test abnormalities on cognitive trajectory group based on CDR-SB score, we performed a logistic regression analysis after entering age (continuous), neuropsychological test abnormalities on each cognitive domain (three categories: language, visuospatial, frontal/executive functions), and single- or multiple-domain involved status as the independent variables, and group as the dependent variable. To determine whether there were significant differences in neuropsychological performance over time between the groups, we also performed linear mixed effects modeling using age, years of education, group, time, and the interaction term between group and time (group by time) as fixed effects, and patient as a random effect. Statistical significance was set at *p* < 0.05 in two-tailed tests. Statistical analyses were performed using SPSS version 20.0 (SPSS Inc., Chicago, IL, USA).
